# Accelerated Silicosis—An Emerging Epidemic Associated with Engineered Stone. Comment on Leso, V. et al. Artificial Stone Associated Silicosis: A Systematic Review. *Int. J. Environ. Res. Public Health* 2019, *16* (4), 568, doi:10.3390/ijerph16040568

**DOI:** 10.3390/ijerph16071179

**Published:** 2019-04-02

**Authors:** Graeme Edwards

**Affiliations:** The Work Doctor, Gold Coast, QLD 4214 Queensland, Australia; drgedwards@workandhealth.com.au

**Keywords:** Silicosis, engineered stone, exposure assessment, surveillance, computed tomography

## Abstract

The systematic review by Leso et al. (16 February 2019) is a timely contribution to the body of knowledge concerning silicosis. It highlights the lack of quality data necessary to inform both occupational health risk management and the clinical management of workers exposed to respirable crystalline silica. This communication highlights current activity being undertaken in Queensland, Australia, that will further inform our knowledge concerning this entirely preventable disease. We are about half-way through a government-funded, case-finding program involving over 800 workers from the engineered stone bench-top fabrication industry. As of 15 February 2019, 99 confirmed cases of silicosis associated with engineered stone work were identified; nearly all were asymptomatic. The empirically observed false negative rate of ILO CXRs in this high-risk group appeared significantly greater than 10%. From pooled data, we hope to develop an appropriate index of exposure to trigger health surveillance using low-dose chest HRCT. Once a worker develops symptoms of silicosis, apart from lung transplantation, there are no treatment options currently available.

The systematic review by Leso et al. (16 February 2019) [1] is a timely contribution to the body of knowledge concerning silicosis. Significantly, most cases reviewed were identified by their clinical presentation to respiratory physicians, with symptoms being indicative of well-established disease. Leso et al. highlighted the lack of quality data to inform both occupational health risk management and the clinical management of workers exposed to respirable crystalline silica years before clinical detection.

The emergence of an epidemic of accelerated silicosis associated with engineered or artificial stone in Australia was recently highlighted by The Lancet [2]. Following inspector activity in Queensland, Australia, triggering health monitoring of workers, I advised the health and safety regulator on 5 September 2018 of 12 cases of silicosis out of all 35 workers examined from just two stone bench-top fabricating businesses. Since then, as of 15 February 2019, a government-funded case-finding program has revealed 99 confirmed cases of silicosis, 15 with progressive massive fibrosis. About half of the 800 workers registered with the program have been screened. My youngest worker is 23 years with a 6-year exposure history.

The reason for this communication is that, at the time of diagnosis of this entirely preventable disease, nearly all workers had minimal or no symptoms, nor did they manifest respiratory function at or below the lower limit of normal for their population-normative reference data. Consistent with the observations of Weissman [3] and others, the screening program in this high-risk group, when reassessed by chest high-resolution computer tomography (HRCT), suggested an International Labor Organisation (ILO) standardized chest radiography (CXR) false negative rate significantly greater than 10%. We are therefore reviewing 16 of the 23 workers from our initial cohort of 35, who had negative ILO CXRs, no symptoms, but significant exposure histories.

Currently, the only index of exposure is ‘years since first exposure’, unqualified by the nature or intensity of exposure. By consensus of the involved clinicians, an exposure threshold has been set to trigger investigation with low-dose HRCT: at least 3 years since first work with dry processes, and dry processing in their work environment constituting at least 25% of 6 months’ work. Any worker with symptoms, or respiratory function at or below the lower limit of population-based normative data, are also assessed by HRCT, even if their exposure history is less than the threshold criteria.

With pooled data analysis, we hope to stratify the exposure histories and develop a better understanding of the cumulative lung burden necessary to trigger disease. We also hope to propose a reasoned ‘index of exposure risk’ to trigger systematic health surveillance with low-dose HRCT, and thus improve health surveillance effectiveness for engineered stone workers and workers in other risk settings, such as mining and quarrying.

As Leso et al. and others [4,5] have reported, once a worker develops symptoms of silicosis, apart from lung transplantation, no other treatment options are currently available. Arising from the current incomplete case-finding activity, if a health surveillance and disease registry of these high-risk workers is established, plausible treatment interventions for this patient population might be demonstrable.
